# ABO blood types and major outcomes in patients with acute hypoxaemic respiratory failure: A multicenter retrospective cohort study

**DOI:** 10.1371/journal.pone.0206403

**Published:** 2018-10-25

**Authors:** Emanuele Rezoagli, Stefano Gatti, Silvia Villa, Giulia Villa, Stefano Muttini, Fabio Rossi, Loredana Faraldi, Roberto Fumagalli, Giacomo Grasselli, Giuseppe Foti, Giacomo Bellani

**Affiliations:** 1 School of Medicine and Surgery, University of Milan-Bicocca, Monza, Italy; 2 Lung Biology Group, Regenerative Medicine Institute (REMEDI) at CÚRAM Centre for Research in Medical Devices, Biomedical Sciences Building, National University of Ireland Galway, Galway, Ireland; 3 Department of Anaesthesia and Intensive Care Medicine, Galway University Hospitals, SAOLTA University Health Group, Galway, Ireland; 4 Department of Emergency Medicine and Intensive Care, "Ospedale Civile" Vimercate, Vimercate, Monza Brianza, Italy; 5 Immunotransfusional Unit, San Gerardo Hospital, Monza, Italy; 6 Department of Anesthesia and Critical Care, ASST Grande Ospedale Metropolitano Niguarda, Milan, Italy; 7 Dipartimento di Fisiopatologia Medico-Chirurgica e dei Trapianti, Università degli Studi di Milano, Milan, Italy; 8 Dipartimento di Anestesia, Rianimazione ed Emergenza Urgenza, Fondazione IRCCS Ca' Granda—Ospedale Maggiore Policlinico, Milan, Italy; 9 Department of Emergency and Intensive Care, San Gerardo Hospital, Monza, Italy; Hospital Universitari Bellvitge, SPAIN

## Abstract

**Introduction:**

ABO blood type A was reported to correlate with an increased risk of acute respiratory distress syndrome (ARDS) in white patients with severe sepsis and major trauma compared with patients with other blood types. Information regarding ABO phenotypes and major outcomes in patients with ARDS is unavailable. The primary aim was to determine the relationship between ABO blood type A and intensive care unit (ICU) mortality in patients with acute hypoxemic respiratory failure (AHRF). The secondary aim was to describe the association between ABO blood type A and ICU length of stay (LOS) in this study population.

**Methods:**

In a multicenter, retrospective cohort study, we collected the clinical records of patients admitted from January 2012 to December 2014 in five ICUs of Northern Italy. We included adult white patients admitted to the ICU who were diagnosed with AHRF requiring mechanical ventilation.

**Results:**

The electronic records of 1732 patients with AHRF were reviewed. The proportion of patients with ABO blood type A versus other blood types was 39.9% versus 60.1%. ICU mortality (25%) and ICU LOS (median [interquartile range], 5 [2–12] days) were not different when stratified by ABO blood type (ICU mortality, overall p value = 0.905; ICU LOS, overall p value = 0.609). SAPSII was a positive predictor of ICU mortality (odds ration [OR], 32.80; 95% confidence interval [CI], 18.80–57.24; p < 0.001) and ICU LOS (β coefficient, 0.55; 95% CI, 0.35–0.75; p < 0.001) at multivariate analyses, whereas ABO blood type did not predict ICU outcome when forced into the model.

**Conclusion:**

ABO blood type did not correlate with ICU mortality and ICU LOS in adult patients with AHRF who were mechanically ventilated.

## Introduction

Acute hypoxemic respiratory failure (AHRF) is a frequent cause of intensive care unit (ICU) admission, requiring positive pressure ventilation and significantly contributing to morbidity and mortality. Its most severe form, acute respiratory distress syndrome (ARDS), is an under-recognized and severe cause of respiratory failure, with a high ICU mortality rate of up to 35% [1]. ARDS is characterized by a local inflammatory response with endothelial activation and microvascular injury contributing to the disruption of the alveolar–capillary barrier. The consequent increased permeability leads to a protein-rich edema fluid into the alveoli, causing hypoxemia [2].

The most common risk factors for ARDS are pneumonia, extrapulmonary sepsis, and aspiration [1]. However, “patient-related” risk factors, such as older age, non-Caucasian race, and specific genetic subsets, are also associated with an increased risk for developing ARDS [3].

Biomarkers of endothelial and alveolar epithelial injury, such as von Willebrand factor (vWF) and soluble intercellular adhesion molecule-1 (ICAM-1), have been associated with pathogenesis [4,5] and outcome [5–7] of patients with acute lung injury.

Clinical studies have suggested that the ABO blood type may influence the risk of both arterial [8–10] and venous vascular thrombosis [11,12], being correlated with different levels of vWF [13,14] and ICAMs [15,16], molecules highly associated with the clinical history of ARDS [4–7].

Reilly et al. recently observed that ABO blood type A is associated with an increased risk for ARDS in white patients with severe sepsis compared with those with other blood types. Similar findings were reported in critically ill white patients hospitalized after major trauma [17].

Based on the findings by Reilly et al., we proposed to understand if ABO blood type could be associated with ICU outcomes in a patient population fulfilling the criteria of acute non-cardiogenic hypoxemic respiratory failure. We performed a retrospective study to investigate whether ABO blood type A was associated with ICU mortality and ICU length of stay (LOS) in a large cohort of critically ill patients with AHRF.

## Materials and methods

This multicenter, observational, retrospective cohort study was conducted according to the Declaration of Helsinki and the Italian guidelines of good clinical practice and Data Protection Code.

Personal information of patients was recorded anonymously using an alpha-numeric code and was filed electronically.

We collected the clinical records of patients admitted from January 2012 to December 2014 in the general ICUs of five different Italian hospitals (San Gerardo Hospital, Monza; Alessandro Manzoni Hospital, Lecco; Vimercate Hospital, Vimercate; Niguarda Ca’ Granda Hospital, Milano; and IRCCS Ospedale Maggiore Policlinico, Milano). The coordinating center was San Gerardo Hospital in Monza. The Ethical Committee of Monza-Brianza Province, ASST Monza–Ospedale San Gerardo–Anestesia e Rianimazione, Monza, Italy (Chairperson Prof. V. Locatelli), approved this study (Ethical Committee N° AB0-ARF (2319)). Informed consent was waived based on the retrospective observational nature of the investigation.

The inclusion criteria were adult white patients and presence of AHRF at admission, defined as a PaO_2_/FiO_2_ < 300 and need for invasive mechanical ventilation with a PEEP of ≥5 [1]. The exclusion criteria were patients admitted to ICU following elective surgery and cardiogenic shock and/or acute cardiogenic pulmonary edema as the primary cause of respiratory failure.

We collected the following clinical data from patient electronic medical records: sex, age, ABO blood type (A, B, AB, and O), Rh blood type (positive or negative), weight, height, simplified acute physiology score II (SAPSII) at admission [18], ICU LOS in patients who survived at discharge, and ICU mortality.

### Statistical analysis

Normality of distribution was assessed using the Shapiro–Wilk test and by visual assessment of variable distribution using histogram. Continuous variables were described as mean ± SD or median (interquartile range, IQR), as appropriate. Binary variables were described as count and proportion (%).

Differences in continuous baseline characteristics between blood type A and other blood types were tested with unpaired Student *t*-test, Mann–Whitney U test, or Chi-squared test.

ICU mortality and ICU LOS were compared between different ABO blood types with the Pearson Chi-squared test and ordinary one-way analysis of variance, respectively. Multiple comparisons *post-hoc* analyses were performed using pairwise Chi-squared tests (ICU mortality) and Bonferroni correction (ICU LOS).

Univariate logistic regression was used to examine the association between survival and blood type A versus other blood types in all patients (primary outcome).

Univariate linear regression was used to examine the association between ICU LOS and blood type A versus other blood types in patients who survived at discharge (secondary outcome).

Variables that were not normally distributed were inserted into the models after log transformation (log).

The association between clinical data, participating centers, and patient outcomes was further tested at the univariate analysis.

Each single variable that correlated with survival or with hospital LOS with p < 0.10 was eligible for inclusion in a multivariate logistic or linear regression, respectively. Backward elimination was performed to finalize the independent predictors of the multivariate models.

The variable defining the presence of blood type A versus other blood types was forced into the multivariate model to explore the study hypothesis. Multicollinearity among predictors of outcomes in the multivariate models was tested using the study of variance inflation factors.

Statistical significance was achieved when p was <0.05 (two-tailed). Statistical analyses were performed using STATA-14/MP (StataCorp LP, College Station, TX, USA) and Microsoft Excel for Mac 2017, Version 15.32.

The study was conducted and reported based on the Strengthening the Reporting of Observational Studies in Epidemiology (STROBE) guidelines for observational studies [19].

## Results

### Baseline characteristics

A cohort of 1732 patients was included in the study. The ABO blood type distribution was blood type A: 39.9% and other blood types: 60.1%. Baseline patient characteristics are summarized in Table 1 and S1 Table.

**Table 1:** Patient baseline characteristics.

	ABO blood type A (n = 691)	ABO blood type non-A (n = 1041)	P-value
Sex, M/F (%)	242/368 (35/65)	449/673 (43/57)	0.888
Age, years, median (IQR)	68 (56–77)	68 (56–77)	0.643
SAPSII, median (IQR)	42 (31–56)	43 (32–59)	0.294
Weight, kg, median (IQR)	75 (65–85)	73 (65–85)	0.595
Height, cm, median (IQR)	170 (163–175)	170 (165–175)	0.623
Rh, n (%)			0.437
• Positive	600 (86.8)	917 (88.1)	
• Negative	91 (13.2)	124 (11.9)	
Admission GCS, median (IQR)	15 (11–15)	15 (10–15)	0.722
Admission Hospital, n (%)			0.832 (overall comparison)
• San Gerardo, Monza	114 (16.5)	152 (14.6)	
• A. Manzoni, Lecco	251 6.3)	390 (37.5)	
• Vimercate Hospital	55 (8.0)	82 (7.9)	
• Niguarda Ca’ Granda, Milano	89 (12.9)	145 (13.9)	
• IRCCS Ospedale Maggiore Policlinico, Milano	182 (26.3)	272 (26.1)	

Abbreviations: n = number; M = male; F = female; IQR = interquartile range; SAPSII = simplified acute physiologic score II; GCS = Glasgow Coma Scale.

### Study outcomes: ICU mortality and ICU LOS

**Fig 1:**
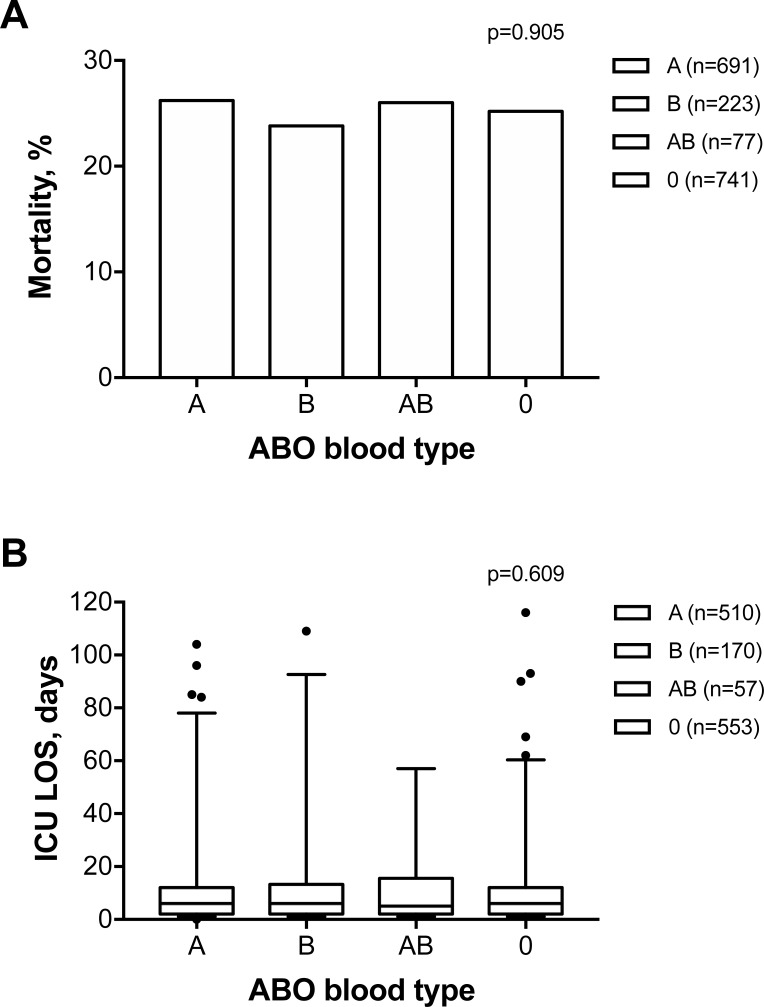
**Frequency of ICU mortality (A) and ICU length of stay in patients who survived at discharge (B) stratified by ABO blood type.** Data in panel **A** are expressed as frequency (%) and in panel **B** as box and whiskers plots (1–99 percentile) with outliers (dots).

More than a quarter (25.5%; n = 441) of patients died in the hospital. No difference in mortality was found among patients with different ABO blood types (ABO blood type, deceased/all patients; frequency: 1. type A, 181/691, 26.2%; 2. type B, 53/223, 23.8%; 3. type AB, 20/77, 26.0%; 4. type 0, 187/741, 25.2%; overall p value = 0.905; Fig 1A).

The median ICU LOS of patients who survived at discharge was 6 days (IQR, 2–12 days) and was not different among patients with different ABO blood types (type A, 6 days [2–12 days]; type B, 6 days [2–13 days]; type AB, 5 days [2–15 days]; type 0, 6 days [2–12]; overall p value = 0.609; Fig 1B).

Major outcomes stratified by the institution of patient admission are summarized in S2 Table and S3 Table.

### Predictors of ICU mortality

In the univariate analysis, two clinical variables at admission were associated with a higher ICU mortality: age (OR, 3.02; 95% CI, 1.94–4.70; p < 0.001) and SAPS II (OR, 32.55; 95% CI, 18.68–56.72; p < 0.001). Higher Glasgow coma score (GCS) score (OR, 0.27; 95% CI, 0.20–0.37; p < 0.001), longer ICU LOS (OR, 0.84; 95% CI, 0.76–0.92; p < 0.001), and higher weight (OR, 0.54; 95% CI, 0.31–0.96; p = 0.035) were associated with lower ICU mortality (Table 2). In the multivariate analysis, only SAPS II retained a significant association with mortality (OR, 32.80; 95% CI, 18.80–57.24; p < 0.001) (Table 3).

**Table 2:** Univariate logistic regression of patient admission variables associated with AHRF ICU mortality.

	OR	*p*	95% CI
Male	1.07	0.539	0.86–1.35
Age	3.02	**<0.001**	1.94–4.70
SAPSII	32.55	**<0.001**	18.68–56.72
Weight	0.54	**0.035**	0.31–0.96
Height	0.99	0.990	0.96–1.04
ABO A-blood type	1.07	0.569	0.86–1.33
Rh positive	0.97	0.833	0.70–1.34
GCS	0.27	**<0.001**	0.20–0.37
ICU LOS	0.84	**<0.001**	0.76–0.92

Abbreviations: SAPSII = simplified acute physiologic score II; GCS = Glasgow Coma Scale; ICU LOS = length of stay in Intensive Care Unit.

**Table 3:** Multivariate logistic regression of patient admission variables associated with AHRF ICU mortality.

	OR	*p*	95% CI
SAPSII	32.80	**<0.001**	18.80–57.24
ABO A-blood type	1.14	0.454	0.80–1.63

Abbreviations: SAPSII = simplified acute physiologic score II.

### Predictors of ICU LOS

In univariate analysis, SAPS II was positively associated with ICU LOS (β coefficient, 0.56; 95% CI, 0.36–0.75; p < 0.001). In contrast, age (β coefficient, −0.67; 95% CI, −0.88 to −0.47; p < 0.001), GCS (β coefficient, −0.40; 95% CI, −0.65 to −0.16; p = 0.001), and male sex (β coefficient, −0.17; 95% CI, −0.31 to −0.04; p = 0.010) were negatively associated with ICU LOS. These results were confirmed by the multivariate analysis for SAPS II (β coefficient, 0.02; 95% CI, 0.01–0.03; p < 0.001) (Table 4 and Table 5). Age and GCS are components of the SAPS II score, and moderate collinearity could not be excluded and were not added to the model.

**Table 4:** Univariate linear regression of admission variables of AHRF patients associated with ICU length of stay in patients who survived at discharge (n = 1291).

	β	*p*	95% CI
Age	-0.67	**<0.001**	-0.88 –-0.47
Male	-0.17	**0.010**	-0.31 –-0.04
ABO A-blood type	-0.02	0.784	-0.15–0.11
Rh positive	-0.03	0.775	-0.22–0.17
SAPSII	0.56	**<0.001**	0.36–0.75
Weight	0.23	0.201	-0.12–0.59
Height	-0.01	0.377	-0.03–0.01
GCS	-0.40	**0.001**	-0.65 –-0.16

Abbreviations: SAPSII = simplified acute physiologic score II; GCS = Glasgow Coma Scale.

**Table 5:** Multivariate linear regression of admission variables of AHRF patients associated with ICU length of stay in patients who survived at discharge (n = 1291).

	β	*p*	95% CI
SAPSII	0.55	**<0.001**	0.35–0.75
ABO A-blood type	-0.03	0.677	-0.20–0.13

Abbreviations: SAPSII = simplified acute physiologic score II.

## Discussion

In this retrospective observational study of a large cohort of patients with AHRF undergoing mechanical ventilation, we did not observe any significant correlation between ABO blood type A with ICU mortality compared with other ABO blood types. Furthermore, ABO blood type A did not predict ICU LOS in our statistical model.

Several studies have shown that ABO blood type is a relevant genetic factor playing a key role in regulating hemostasis [12,13,20,21]. Furthermore, two meta-analyses of the literature reported that the non-O blood group was a potential genetic risk factor for venous thrombosis [13], whereas patients with blood type O group had a higher risk of bleeding compared with those with non-O blood type [21]. Non-O blood group individuals have higher levels of vWF [22] and higher vWF adhesive activity [23]. vWF may be a useful biomarker in identifying patients with ARDS at higher risk for mortality [24–26] and prolonged ventilation [26,27]. Despite the reported association between ABO blood type A and the risk for developing ARDS, we did not observe any correlation between ABO blood group and relevant clinical outcomes in our patient population. Our results were obtained from a heterogeneous population of patients with AHRF from different causes, and we cannot exclude an association between ABO blood type and clinical outcomes in specific subgroups of patients with ARDS, as reported by Reilly et al. for patients with severe sepsis and major trauma.

We observed a significant correlation among SAPS II, age, and GCS with major outcomes in our population, at univariate analysis. These correlations were confirmed for SAPS II in the multivariate models. SAPS II is a robust and validated score, which provides an estimate of the risk of death in a generalized population of medical and/or surgical ICU patients [18]. Of note, the mortality in our population (25.5%) matched the predicted mortality estimated by Le Gall et al., considering a median SAPS II score of 43 in our patients. Older age is accounted among the non-modifiable factors associated with hospital mortality from ARDS, as reported by Laffey et al., in a secondary analysis of the LUNG SAFE study [28]. Furthermore, GCS score is a potentially useful predictor of mortality in general intensive care patients [29].

Interestingly, at the univariate analysis, weight tended to be negatively correlated with mortality, but this finding was not confirmed at the multivariate analysis. Despite the lack of a clear pathophysiologic mechanism, obesity has been associated with lower mortality in patients with ARDS [30], the so-called “obesity paradox” [31]. However, obese patients are prone to develop collapse of the lung-dependent zones by the mechanical weight of the abdominal content, which displaces the diaphragm toward the lung bases [32,33]. Although this mechanism leads to atelectasis-correlated hypoxemia [32] because of a right shift of the pressure–-volume relationship of the respiratory system [33], it can be easily reversed by a recruitment maneuver followed by PEEP [32].

This study has several limitations. First, although we analyzed a large cohort of patients with AHRF, we acknowledge the disadvantages of the retrospective nature of the study. Second, we do not have information on the actual presence of ARDS in these patients and of its severity [1], etiology [34], and phenotype [35]. Hence, we cannot exclude an association between ABO blood type and patient outcome in different subcategories of ARDS. Third, we included only patients with AHRF requiring mechanical ventilation in the study; thus, we cannot disclose a possible role of ABO blood type in patients with mild hypoxemia managed only with non-invasive ventilation. In contrast, the strength of this study is that we collected complete data on survival and ABO blood group in 1732 patients with AHRF, with 40% ABO blood type A. Considering a level of significance (α) of 0.05 (two-tailed), we had a power (1-β) of 0.80 to estimate a difference in intra-hospital mortality as low as 7% between patients with blood type A and those with other blood types.

## Conclusions

In a large multicenter, retrospective cohort study of adult patients on mechanical ventilation for AHRF, ABO blood type did not correlate with ICU mortality and ICU LOS. Future large prospective studies are required to confirm our findings, also considering specific subcategories of patients with ARDS.

## Supporting information

S1 Table Patient baseline characteristics. (DOC)

S2 Table Frequency of mortality in patients who survived at discharge stratified by hospital admission. Pearson’s chi-squared was used to test the overall difference in mortality among different institutions.
(DOC)

S3 Table Frequency ICU length of stay in patients who survived at discharge stratified by hospital admission. Ordinary one-way ANOVA was used to test the overall difference in ICU length of stay among different institutions. Post-hoc comparisons were performed using Bonferroni’s correction. *p<0.05 versus Monza. Abbreviations: IQR = interquartiles.
(DOC)
