# Supplementary material for: ABO blood types and major outcomes in patients with acute hypoxaemic respiratory failure: A multicenter retrospective cohort study
Source: PLoS One. 2018 Oct 25;13(10):e0206403. doi: 10.1371/journal.pone.0206403 (PMC6201964; doi:10.1371/journal.pone.0206403)
Supplement: S1 Table — (DOC) [file pone.0206403.s001.doc]

**S1 Table. Patient baseline characteristics**

| Total number, n | 1732 |
| --- | --- |
| Sex, M/F | 610/1122 |
| Age, years, median (IQR) (n=1649) | 68 (56-77) |
| SAPSII, median (IQR) (n=923) | 43 (32-57) |
| Weight, kg, median (IQR) (n=1133) | 75 (65-85) |
| Height, cm, median (IQR) (n=166) | 170 (165-175) |
| ABO blood type, n (%) |  |
| - A | 691 (39.9) |
| - B | 223 (12.9) |
| - AB | 77 (44.4) |
| - 0 | 741 (42.8) |
| - non-A | 1041 (60.1) |
| Rh, n (%) |  |
| - Positive | 1517 (87.6) |
| - Negative | 215 (12.4) |
| Admission GCS, median (IQR) (n=969) | 15 (11-15) |
| Admission Hospital, n (%) |  |
| - San Gerardo, Monza | 266 (15.4) |
| - A. Manzoni, Lecco | 641 (37.0) |
| - Vimercate Hospital | 137 (7.9) |
| - Niguarda Ca’ Granda, Milano | 234 (13.5) |
| - IRCCS Ospedale Maggiore Policlinico, Milano | 454 (26.2) |

Patients baseline characteristics are described in all patients (n=1732) unless otherwise specified. Abbreviations: n=number; M=male; F=female; IQR=interquartile range; SAPSII=simplified acute physiologic score II; GCS=Glasgow Coma Scale.
