# Supplementary material for: ABO blood types and major outcomes in patients with acute hypoxaemic respiratory failure: A multicenter retrospective cohort study
Source: PLoS One. 2018 Oct 25;13(10):e0206403. doi: 10.1371/journal.pone.0206403 (PMC6201964; doi:10.1371/journal.pone.0206403)
Supplement: S2 Table — Pearson’s chi-squared was used to test the overall difference in mortality among different institutions. (DOC) [file pone.0206403.s002.doc]

**S2 Table. Frequency of mortality in patients who survived at discharge stratified by hospital admission**

|  | Intra-hospital mortality (p=0.150) |
| --- | --- |
| Admission hospital, n (%) |  |
| - Monza (n=266) | 78 (29.3) |
| - Lecco (n=641) | 175 (27.3) |
| - Vimercate (n=137) | 31 (22.6) |
| - Niguarda (n=234) | 50 (21.4) |
| - Policlinico (n=454) | 107 (23.6) |

Pearson’s chi-squared was used to test the overall difference in mortality among different institutions.
