# Supplementary material for: ABO blood types and major outcomes in patients with acute hypoxaemic respiratory failure: A multicenter retrospective cohort study
Source: PLoS One. 2018 Oct 25;13(10):e0206403. doi: 10.1371/journal.pone.0206403 (PMC6201964; doi:10.1371/journal.pone.0206403)
Supplement: S3 Table — Ordinary one-way ANOVA was used to test the overall difference in ICU length of stay among different institutions. Post-hoc comparisons were performed using Bonferroni’s correction. *p<0.05 versus Monza. Abbreviations: IQR = interquartiles. (DOC) [file pone.0206403.s003.doc]

**S3 Table. Frequency of ICU length of stay in patients who survived at discharge stratified by hospital admission**

|  | ICU LOS (p=0.014) |
| --- | --- |
| Admission hospital, days, median (IQR) |  |
| - Monza (n=188) | 7 (4-13) |
| - Lecco (n=466) | 4 (1-12)^*^ |
| - Vimercate (n=106) | 8 (5-17) |
| - Niguarda (n=184) | 8 (3-13) |
| - Policlinico (n=346) | 5 (2-11)^*^ |

Ordinary one-way ANOVA was used to test the overall difference in ICU length of stay among different institutions. Post-hoc comparisons were performed using Bonferroni’s correction. *p<0.05 versus Monza. Abbreviations: IQR=interquartiles.
